# Malignant transformation of Madelung’s disease in a patient with a coincidental diagnosis of breast cancer: a case report

**DOI:** 10.1186/1746-1596-7-116

**Published:** 2012-09-01

**Authors:** Maddalena Borriello, Alessandro Lucidi, Arnaldo Carbone, Vito Iannone, Gabriella Ferrandina

**Affiliations:** 1 Department of Oncology, Gynecologic Oncology Unit, Catholic University, Campobasso, Italy; 2 Gynecologic Oncology Unit, Catholic University, Rome, Italy; 3 Department of Pathology, Catholic University, Campobasso, Italy

**Keywords:** Madelung disease, Malignant degeneration, Breast cancer

## Abstract

**Abstract:**

Madelung’s disease or multiple symmetric lipomatosis (MSL) is a rare disorder of unknown etiology which typically presents symmetrically subcutaneous accumulation of non-encapsulated adipose tissue which slowly grows around the neck, upper part of the arms, pelvis, back and thigh. This disease is also frequently associated with hepatopathy, glucose intolerance, hyperuricemia, and malignant tumors of the upper airways. Nevertheless, only one description of malignant transformation of Madelung’s disease has been presented in literature. Here, we report a case of liposarcomatous transformation of Madelung’s disease in a 59-year-old Italian woman with a coincidental diagnosis of breast cancer.

**Virtual slide:**

The virtual slide for this article can be found here: http://www.diagnosticpathology.diagnomx.eu/vs/3480884087499351

## Background

Multiple symmetric lipomatosis (MSL) also known as Madelung disease or Launois-Bensaude syndrome is a rare disorder of unknown etiology first described in 1846 by Brodie and in 1888 by Madelung [1]. MSL mostly occurs in Mediterranean middle-age male (30–60 years old) with a history of chronic alcoholism [2]. Typical features of this condition are due to symmetric subcutaneous accumulation of non-encapsulated adipose tissue, which slowly grows around the neck, upper part of the arms, pelvis, back and thigh. Enzi et al. [2] described two types of lipomatosis based on the distribution of fat tissue: type I is characterized by lipomas located in the nape of the neck, the supraclavicular and deltoid regions (Madelung’s collar), while in Type II lipomatosis, fat tissue diffuses extensively in to the subcutaneous fat layer giving the patient and appearance of simple obesity. Besides the disfiguring aesthetic effects, in some cases lipomatous masses can infiltrate spaces between adjacent structures, thus leading to signs/symptoms of compression/infiltration of the upper aerodigestive tract (dyspnea, dysphonia, dysphagia), low mobility of the neck, mediastinal involvement, superior vena cava syndrome etc.

The exact pathogenesis is unclear although several theories have been proposed: in particular, enzymatic defects of lipid metabolism (especially for catecholamine-induced lipolysis) and, more recently, the occurrence of mutations of mitochondrial DNA have been involved in the marked increase of adipose tissue. Moreover, some evidences have suggested that cellular elements involved in MSL pathogenesis might be represented by precursors from brown fat tissues [2,3].

There are no clear guidelines for the overall management and treatment of MSL: patients are usually triaged to surgical removal of large lipomas even though medical treatment aimed at correcting metabolic disorders are also employed [3,4]. Besides the association with other pathological conditions such as polyneuropathy and metabolic disturbancies, Madelung’s disease has been also related to the occurrence of oro-pharyngeal tumors [5]; whether these findings are just coincidental or reflect the increased prevalence of risk factors (i.e. alcohol, smoking) in these patients remains unclear [3,5]. On the other hand, only one case of liposarcomatous progression of Madelung’s disease has been reported in the literature [6].

Here, we report a case of malignant transformation of Madelung’s disease in a 59-year-old Italian woman with a coincidental diagnosis of breast cancer.

## Case presentation

In October 2011, a 59-year-old woman was referred to our Institution because of the documentation of a breast nodule suspicious for malignancy.

Her familial history was unremarkable for neoplasias and congenital disorders. The patients reported a >30 year history of alcohol abuse as well as severe gastro-duodenal ulcer, and recurrent episodes of phlebitis of the lower limbs requiring surgery. She also reported multiple surgeries due to large lipomatous masses located in the neck, shoulder and upper arms. She was 147 cm tall and weighted 70 kg (BMI = 32.4).

**Figure 1:**
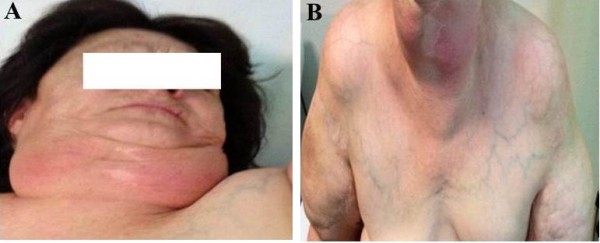
Picture of the patient showing Type I MSL with symmetric accumulation of lipomatous tissue in the anterior cervical region (A) and upper arms (B)

Physical examination showed an enlargement of the anterior cervical region, as well as the presence of symmetric masses of the upper arms typical of Type I Madelung’s disease (Figure 1A).

Patient’s haematological and chemical profile was within normal limits with the exception of elevated serum levels of aspartate (240 IU/l) and alanine (108 IU/l) aminotransferase. Bilirubin and γ-glutamyl-trasferase levels were also above the normal limits (1.33 mg/dl, and 334 IU/l, respectively).

Needle aspiration cytology of a 2 cm breast nodule of the left subareolar region was performed, and was suggestive of carcinoma. Staging work-up, including chest x-rays, US liver examination and PET-CT scan. Ca15-3 and Ca125 levels were within the normal limit.

**Figure 2:**
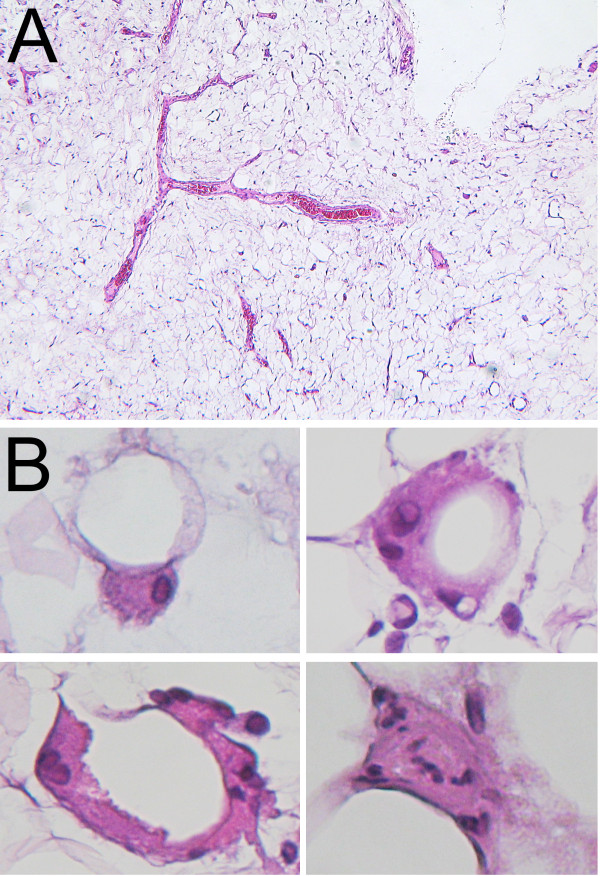
Malignant transformation of axillary lipomatous masses in Madelung’s disease. **A**) At low magnification vascular density was evident with abundance of vessels with characteristic arborizing shapes (H&E; original magnification: 200x). **B**) Lipoblasts were frequently observed at high power magnification (insets), together with the typical delicate vascular network (not shown). In the lower right inset an atypical mitosis is shown. These findings indicated a well differentiated liposarcoma (H&E; original magnification: 400x)

In November 2011 the patient underwent total left mastectomy and sentinel lymph node biopsy. Frozen section analysis was positive for carcinoma both in the breast and in the sentinel lymph node. Therefore, a cautious axillary lymph node dissection was performed after a difficult identification of the axillary vein which appeared intermingled within an apparently lipomatous mass. Final histology showed invasive breast carcinoma with a poor grade of differentiation and metastatic lymph node involvement (final stage: pT1cN1M0). Histopathologic analysis of resected axillary fat tissue showed a well differentiated liposarcoma with myxoid aspects. In particular, no signs of flogosis was observed, while the lipoblasts are characterized by the morphology of the neoplastic elements with an atypical nucleus containing a nucleolus, and a cytoplasm with a single, large lipidic vacuole (Figure 2 A,B). No immunohistochemistry was required for the diagnosis.

A multidisciplinary team including Medical Oncologists and Internists carefully evaluated the need to administer adjuvant treatment with the presence of comorbidities: indeed, based on stage and grade of breast cancer, the documentation of positive Ki67 staining in 42% of tumor cells as well as the concomitant diagnosis of liposarcoma, the patient was triaged to chemotherapy with single agent epirubicine followed by hormone treatment.

## Conclusions

To our knowledge, this is the second case reported in the literature of liposarcomatous transformation occurring in a patient affected by Madelung’s disease. The first description dates back to 1983, when Tizian et al. [6] reported on a 57-year-old woman with a 7-year history of progressive, symmetric thickening of the neck due to histologically confirmed benign lipomatosis. Changes of clinical appearance of the left side of neck lipomatosis, which had become smaller, harder and fixed to the skin, as well as the occurrence of dyspnoea and limited mobility of the cervical spine had accelerated the execution of radiographic studies which confirmed infiltration of soft tissues, but failed to document any tumor mass displacing the trachea. After surgical resection of lipomatous masses, histopathology had revealed the presence of areas of myxoid liposarcoma in the context of larger parts of embrional lipoblasts, and the patient had been triaged to adjuvant chemotherapy [6].

In our MSL case, the diagnosis of liposarcomatous progression was documented in the left axillary lipomas removed because of a coincidental diagnosis of breast cancer: indeed, at clinical examination, no suspicion of axillary tumor involvement had emerged; moreover, staging work up with US, mammography, and PET/CT scan also failed to document any abnormalities. CT and MRI have been reported to play a relevant role in assessing the extension and distribution of lipomatous masses [7], however, the difficulties to differentiate benign lipomas versus liposarcoma solely on radiological basis are also acknowledged [8]. Therefore, any modification of lipomatous masses, especially if asymmetric, and the appearance of signs/symptoms of compression/infiltration of aerodigestive tract should be taken into account for planning surgery which still remains the mainstay of treatment. A prompt removal of lipomatous masses confirms histology, increases the chances of a radical excision, and could also help to diagnose malignant airway tumors whose symptoms could go underestimated because attributed to a long standing Madelung’s disease [5].

On the other hand, in cases refusing surgery or still considered as not requiring surgical removal of asymptomatic masses, a biopsy could be performed even though the diagnostic limits inherent in this procedure should be recognized.

No clear guidelines for the overall management of Madelung’s disease are available given the rarity of disease: removal of lipomatous masses by lipectomy, or US-assisted liposuction in selected cases [9] is strongly recommended. Recently, also intralesional injections of phosphatidylcholine (mesotherapy) has been reported as a potentially effective therapy for MSL [10].

Moreover, regular follow up visits could be advocated for early diagnosis of aerodigestive tract malignancies or liposarcomatous transformation. In fact, malignant transformation of lipomas documented by us and Tizian et al. [6] correspond to 0.6-0.7% of cases with Madelung’s disease reported in the literature (2 out of approximately 300 cases). A more in-depth analysis of molecular pathways associated with MSL would open novel perspectives in terms of disease management and understanding of pathogenesis: indeed, MSL adipocytes have been proposed to originate from the brown adipose tissue precursors given the similarity of morphological appearance and expression of specific molecular markers, such as the mitochondrial inner protein UCP-1 [11]. Moreover, MSL adipocytes exhibit an increased, so-called *tumor-like,* proliferation rate, and have been described as showing intermediate morphological features between lipoma and liposarcoma [12]. Whether the mutations of mitochondrial DNA described in MSL adipocytes are directly responsible of the increased growth rate [13] or are just an epiphenomenon of disturbances of other metabolic pathways remains to be established.

## Consent

Written informed consent was obtained from the patient for publication of this case report and any accompanying images. A copy of the written consent is available for review by the Editor-in-Chief of this Journal.

## Competing interests

The authors declare that they have no competing interests.

## Authors’ contribution

MB: conceived the study, and was in charge of surgery; AL: participated in manuscript drafting; AC: carried out the histopathological evaluation, and helped in drafting the manuscript; VI: was involved in surgery and manuscript drafting; GF: coordinated the study, and drafted the manuscript. All Authors read and approved the final manuscript.
